# Renal denervation restores biomechanics of carotid arteries in a rat model of hypertension

**DOI:** 10.21203/rs.3.rs-3273236/v1

**Published:** 2023-09-05

**Authors:** Anastasia Gkousioudi, Margherita Razzoli, Jesse D. Moreira, Richard D. Wainford, Yanhang Zhang

**Affiliations:** Boston University; Boston University; Boston University Avedisian and Chobanian School of Medicine; Boston University Avedisian and Chobanian School of Medicine; Boston University

## Abstract

The prevalence of hypertension increases with aging and is associated with increased arterial stiffness. Resistant hypertension is presented when drug treatments fail to regulate a sustained increased blood pressure. Given that the mechanisms between the sympathetic nervous system and the kidney play an important role in blood regulation, renal denervation (RDN) has emerged as a therapeutic potential in resistant hypertension. In this study, we investigated the effects of RDN on the biomechanical response and microstructure of elastic arteries. Common carotid arteries (CCA) were excised from 3-, 8- and 8-month-old denervated rats, and subjected to biaxial extension-inflation test. Our results showed that hypertension developed in the 8-month-old rats. The sustained elevated blood pressure resulted in arterial remodeling which was manifested as a significant stress increase in both axial and circumferential directions after 8 months. RDN had a favorable impact on CCAs with a restoration of stresses in values similar to control arteries at 3 months. After biomechanical testing, arteries were imaged under a multi-photon microscope to identify microstructural changes in extracellular matrix (ECM). Quantification of multi-photon images showed no significant alterations of the main ECM components, elastic and collagen fibers, indicating that arteries remained intact after RDN. Regardless of the experimental group, our microstructural analysis of the multi-photon images revealed that reorientation of the collagen fibers might be the main microstructural mechanism taking place during pressurization with their straightening happening during axial stretching.

## Introduction

Arterial stiffness of elastic arteries depends mainly on the fibrous extracellular matrix (ECM) components, namely collagen and elastic fibers, and their organization. Elastic and collagen fibers are the two major ECM constituents in large elastic arteries and the primary load-bearing ECM components of the arterial wall. Elastic fibers provide arteries with the ability to accommodate pulsatile blood flow and collagen fibers primarily provide structural integrity and distensibility. Collagen has a half-life of ~ 22 days in arteries (1). In contrast, elastin is only produced in early development and childhood, has a very low turnover rate, and provides elasticity to the tissue through the lifetime of the individual (2).

Hypertension is the most common cardiovascular disease affecting around 1 billion people worldwide and its prevalence increases with age (3). Hypertension has long been associated with arterial stiffening (4, 5). Vascular cells sense the elevated blood pressure and respond with an abnormal metabolic activity and ECM protein production which results to an accelerated vascular stiffening (6). Remodeling of vascular ECM in hypertension is manifested with collagen deposition and elastin fragmentation which alters the collagen-to-elastin ratio and results in arterial wall thickening, while the expression of proteoglycans and glycoproteins is also upregulated (5, 7–9). Several studies have reported stiffening of the arterial wall of systemic arteries in hypertension in both clinical and animal studies (10–13). Therefore, there is a strong connection between arterial stiffness and hypertension.

Resistant hypertension manifests when the conventional antihypertensive therapy fails to control the blood pressure. This persistent and prolonged high blood pressure carries a mortality risk to resistant hypertensive patients who are more likely to develop irreversible damage in end-organs such as the kidneys and brain due to cardiovascular implications (14). Several studies have reported the role of the renal sympathetic nervous system, which is comprised of both afferent and efferent nerves, as a regulatory mechanism for the blood pressure through sodium, renin and renal blood flow modulation (15–17). Previous studies provided a wealth of evidence supporting a key role of increased sympathetic outflow in hypertension. Renal denervation (RDN) has been emerged as a potential therapeutic approach to resistant hypertension by selectively removing the renal nerves and therefore attenuating renal sympathetic outflow. Clinical trials have been conducted to study the effects of RDN in resistant hypertensive patients, although inconsistent outcomes failed to quantitatively assess the effects of the procedure (18). However, to the best of our knowledge, none of previous studies investigated the effect of RDN on microstructure and biomechanical response of elastic arteries.

This study focuses on understanding the interplay between sympathetic outflow and vascular remodeling. A rat model was used to study the remodeling and mechanical response of elastic arteries in hypertension and RDN. Common carotid arteries (CCA) were harvested from 3-, 8- and 8-month-old denervated rats and subjected to biaxial inflation-extension testing; circumferential and axial stresses as well as tangent modulus were calculated. Arterial ECM were investigated through multi-photon microscopy and microstructural parameters (waviness and orientation) of collagen and elastic fibers were extracted. The combined results from mechanical testing and multiphoton imaging allowed us to assess the structure and function of CCA in hypertension and RDN.

## Materials and Methods

### Animals

Male Sprague-Dawley rats aged 3-, and 8-months old were purchased from Envigo (Indianapolis, IN, USA). Rats were pair-housed prior to surgical intervention and were individually housed following survival surgery as described below, in accordance with Animal Research: Reporting of In Vivo Experiments (ARRIVE) guidelines. Animals were housed in a temperature (range 68–79°F) and humidity (range 30–70%) controlled facility under a 12-h light-dark cycle and were allowed tap water and standard irradiated normal salt rodent diet (NS; Envigo Teklad, WI, Teklad Global Diet #2918, 18% protein, 5% crude fat, 5% fiber, total potassium (K^+^) content 0.6%, total NaCl content 0.6% [174 mEq Na^+^/kg]) *ad libitum*. In all studies rats were randomly assigned to experimental groups. All animal protocols were approved by the Institutional Animal Care and Use Committee under protocol number PROTO201800201 in accordance with the guidelines of the Boston University Chobanian & Avedisian School of Medicine and the National Institutes of Health *Guide for the Care and Use of Laboratory Animals*. All possible steps were taken to minimize pain and suffering, and euthanasia was conducted in accordance with approved protocols.

### Surgical Procedures

#### Acute femoral vein, artery, and bladder cannulation

Rats were anesthetized (sodium brevital, 20 mg/kg IP, supplemented with 10 mg/kg IV as required) and the left femoral vein, left femoral artery, and bladder were cannulated with PE-50 (vein and artery) or PE-240 (bladder) tubing to deliver intravenous infusions, measure arterial blood pressure, and collect urine, respectively. Rats were then gently placed in a Plexiglas holder and received an intravenous infusion of isotonic saline (20 µL/min) during a 2-h recovery period prior to experimentation for the stabilization of prior to experimentation for the stabilization of cardiovascular and renal excretory functions and to reach full consciousness. Mean arterial pressure (MAP) and heart rate (HR) were recorded continuously via the femoral artery cannula using BIOPAC data acquisition software (MP150 and AcqKnowledge 3.8.2; BIOPAC Systems) in conjunction with an external pressure transducer (P23XL; Viggo Spectramed) (19–23).

#### Bilateral Renal Denervation (RDNX)

In 8-month-old male rats, RDNX was performed for 14 days prior to acute blood pressure measurement. In these animals, standard techniques were used to remove the influence of both the afferent and efferent renal sympathetic nerve fibers (21, 24–26). In brief, under sodium pentobarbital anesthesia (30 mg/kg IP), each kidney was exposed by a dorsal flank incision. Using a dissecting microscope, the renal vein and artery were dissected from the surrounding fascia and all visible renal nerve bundles were removed. After dissection, the renal artery was coated with a 10% phenol solution in ethanol to ensure the destruction of any remaining renal nerve fibers. In the sham denervation group, each kidney was exposed, and the renal artery and vein were visualized prior to suturing. The effectiveness of RDNX was confirmed at the end of the blood pressure measurement study by ELISA analysis of NE content in kidney tissue as per manufacturer’s instructions (ELISA IB89537, IBL America, MN) (26). In intact 8 months old rats renal NE content was 832 ± 34 pg/mg and was reduced to 58 ± 18 pg/mg following renal denervation.

#### Cardiovascular function

After a 2-h recovery period, baseline MAP was continuously recorded in naive conscious rats via the femoral artery cannula during a 1-h blood pressure measurement period. Reported MAP values represent the average MAP during the entire 1-h period (19, 27). Hypertension was developed in the 8 months group with a MAP of 136.2 ± 0.8 mmHg, which is significantly higher than the 3 months group with a MAP of 124.8 ± 0.8 mmHg (p < 0.05; Figure S1A). RDN attenuated hypertension in 8 months old rats with a MAP of 126.8 ± 1.2 mmHg, a significant decrease from the 8-month group (p < 0.05). Throughout this study, the 3 months old rats are denoted as “control” (CTL), the 8 months old rats that developed hypertension are denoted as “hypertensive” (HT) and the 8 months old rats that underwent renal denervation are denoted as “denervated” (RDN). Nine CCAs were used in each experimental group (n = 9). For some animals, both the left and right CCA were used.

##### Biaxial extension-inflation testing:

The mechanical testing protocol was described in details in our previous study (28). Briefly, the collected arterial samples were cleaned from surrounding tissue and fat, and cannulated on a pressure myograph (110P DMT, Danish Myo Technology, Denmark) to perform the biaxial extension-inflation test. First, *in vivo* axial stretch ratio was estimated as the axial stretch at which the axial force remains constant during pressurization. Prior to testing, arteries were preconditioned to ensure consistent behavior during mechanical testing (29) by stretching axially to their estimated *in vivo* axial stretch ratio, and inflating from 0 to 140 mmHg three times. The mechanical testing protocol included a stepwise inflation from 0 to 120 mmHg (increment of 10 mmHg and a loading rate of 5 mmHg/sec) at three different axial stretch ratios: *in vivo* stretch ratio, and 5% above and below this ratio. Arteries were fully immersed into Krebs-Ringer biocarbonate buffer solution at room temperature (22° C) throughout the testing. Deformed outer diameter, axial force and applied pressure were collected continuously. After the testing, the unloaded arterial dimensions were measured by imaging thin arterial rings (~ 0.5 mm in length; Figures S1B-D). Images were imported into FIJI (http:/Fiji.sc/Fiji, Ashburn, VA) and the outer and inner circumference were manually traced.

Assuming that the artery behaves as a thin-walled cylinder, the mean biaxial Cauchy stresses were calculated as:

(1) $$\sigma_{\theta }\;=\;\frac{{\mathrm{Pr}}_{i}}{h}\;\mathrm{and}\;\sigma_{z}\;=\;\frac{f+P\pi r_{i}^{2}}{\pi h\left(2r_{i}+h\right)}$$

where ***P*** is the transmural pressure, ***h*** is the wall thickness, ***r***_***i***_ is the deformed inner radius and ***f*** is the axial force. Subscripts ***θ*** and ***z*** correspond to circumferential and axial direction, respectively. The arterial wall was assumed to be incompressible; thus, the deformed inner diameter, ***d***_***i***_ was calculated as:

$$d_{i}\;=\;\sqrt{d_{o}^{2}\;-\;\frac{D_{o}^{2}-\;D_{i}^{2}}{\lambda_{z}}}$$

where ***d***_***o***_ is the deformed outer diameter, ***D***_***i***,***o***_ is the undeformed inner and outer diameter and ***λ***_***z***_ is the axial stretch ratio.

#### Multi-photon imaging

The underlying microstructure of the arterial ECM was investigated using a Carl Zeiss LSM 710 NLO multi-photon microscope system equipped with a tunable femtosecond IR pulse laser (Coherent Chameleon Vision-S). Samples were mounted into a custom-made tissue stretching-inflation chamber which allows the biaxial deformation of the artery while being imaged under a microscope (30). Each artery was inflated with phosphate buffered saline (PBS) using a pressure gauge while being immersed into a chamber filled with PBS at a room temperature. Samples were stretched to their *in vivo* stretch ratio and imaged under different pressure values (0, 30, 60, 90 and 120 mmHg). An excitation wavelength of 810 nm was used to generate two-photon excitation of fluorescence (500/550 nm) from elastin and second harmonic generation (395/415 nm) from collagen (30, 31). Arterial samples were imaged to a depth of ~ 50–60 µm with a 1 µm spacing and a field of view of µm using a water immersion objective (20, NA 1.0, W Plan-Apochromat, Zeiss). Z-stacks of elastin and collagen images were acquired at every pressure level.

#### Straightness parameter of collagen fibers

The straightness parameter, of collagen fibers was calculated using CT-FIRE (32), which extracts structural parameters of fiber-like elements. Pre-processing of the image includes the application of a curvelet transform-based denoising filter. Fibers are traced by applying the Euclidean distance transform to the image and to identify nucleation points for fibers. Every image of the collagen z-stack was imported to CT-FIRE and was calculated for the individual traced fibers in the image. A histogram of was generated for every image; all histograms of the collagen stack were combined to a three-dimensional (3-D) bar plot. This procedure was repeated for all pressure levels and axial stretches.

##### Orientation of collagen and elastic fibers:

The acquired Z-stacks of elastic and collagen fiber images were imported to FIJI (http:/Fiji.sc/Fiji, Ashburn, VA) and the fiber orientation was extracted for every image in the stack using the Directionality plug-in. The program uses Fast Fourier Transform (FFT) to obtain the spatial frequencies of the image. The direction of the fibers is extracted by calculating the angle and intensity of each spatial frequency in the FFT power spectra using edge-detector filters. The distribution functions of the stack were averaged and plotted for every pressure. On the other hand, to show the transmural variation in orientation for elastic fibers in the adventitial and outer medial layer, the distribution functions at each imaging depth were combined into a two-dimensional (2-D) surface plot.

#### Histology

After mechanical testing and imaging, an arterial ring of ~ 0.5 mm in length was cut from the center of the artery, fixed in 4% paraformaldehyde (PFA) and stored in 70% ethanol. Samples were then imbedded in paraffin and stained with Movat’s Pentachrome (MOV) to identify collagen, elastin and smooth muscle cells. Histology slides were imaged using an optical microscope with a 40 objective (Olympus VS120 Automated Slide Scanner) at the Micro/Nano Imaging (MNI) Facility at the Biomedical Engineering Core Facilities, Boston University.

#### Statistical analysis

Average experimental data from mechanical testing are presented as mean ± standard error of the mean (SEM) for the biomechanical analysis, and as mean ± standard deviation (SD) for the multi-photon image analysis. A one-way analysis of variance (ANOVA) was used to access statistical differences between the three groups. Post hoc analysis performed using Bonferroni test. A value of p < 0.05 was considered significant. All statistical analyses were performed using IBM SPSS (Version 27.0).

## Results

**Figure 1:**
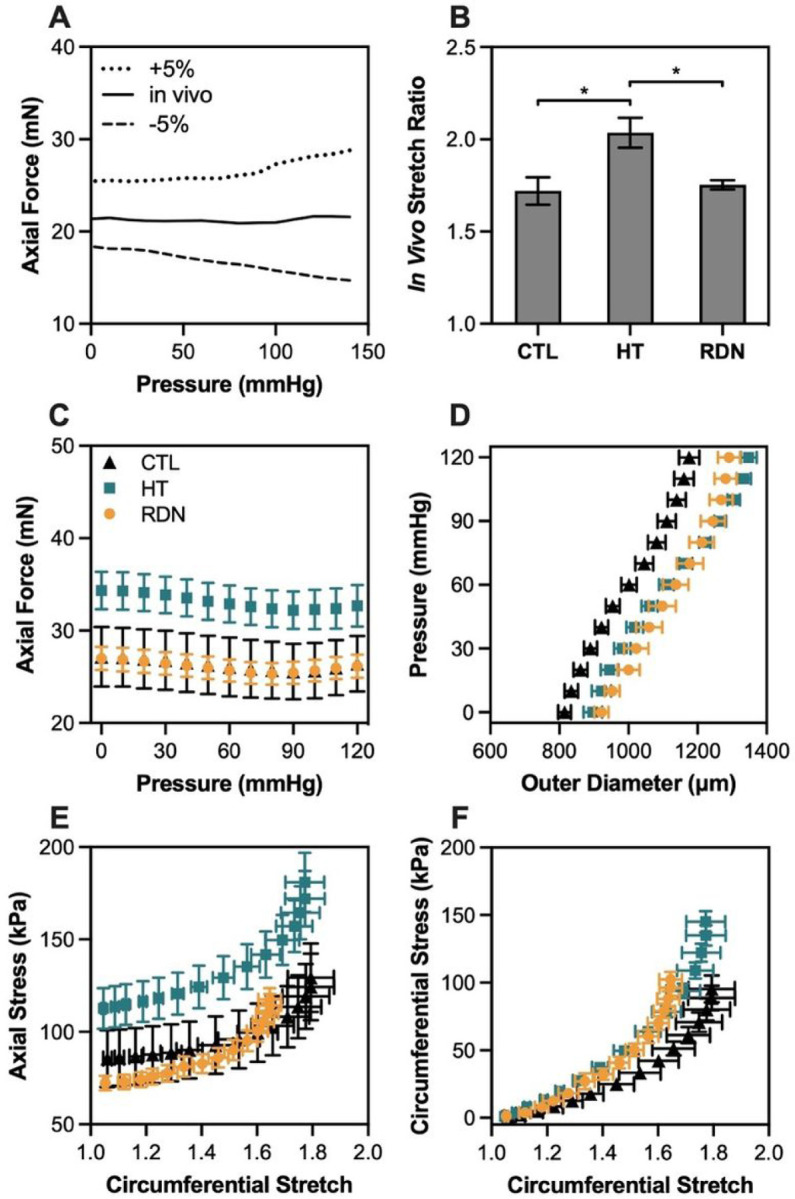
A: Representative axial force-pressure response at three different axial stretch ratios (*in vivo* ± 5%). B: Average (n=9) estimated *in vivo* stretch ratio. C, D: Average (n=9) axial force-pressure and pressure-outer diameter response at *in vivo* axial stretch ratio. E, F: Average (n=9) Cauchy stress-stretch behavior in the axial and circumferential directions. Control, hypertensive and denervated group are denoted as CTL, HT and RDN, respectively. Average values are presented as mean ± SEM (*p<0.05).

Figure 1A shows the axial force at the *in vivo* and ± 5% axial stretch ratio for a representative sample. When the artery was stretched at its estimated *in vivo* axial stretch ratio, the axial force remained constant under pressurization; when the axial stretch ratio increased 5%, the axial force also increased, whereas for a 5% decrease in axial stretch ratio, the axial force decreased with pressure. Figure 1B shows the estimated *in vivo* axial stretch ratio of CCAs for the three experimental groups. Hypertension had a significant impact on *in vivo* stretch ratio which increased from 1.72 ± 0.07 to 2.04 ± 0.08 (p < 0.05) for the 3- and 8-month groups, while RDN resulted in a decrease of the *in vivo* ratio to 1.75 ± 0.02 (p < 0.05).

The pressure-diameter and axial force-pressure data are given in Figs. 1C-F along with the calculated Cauchy stresses in the circumferential and axial directions. The axial force needed to maintain CCAs at their *in vivo* stretch (Fig. 1C) increased with hypertension for the applied pressure range. After RDN, the axial force decreased and reached values similar to the 3-month group. On the other hand, outer diameter of CCAs during inflation seemed to be affected only due to hypertension, not RDN (Fig. 1D). Specifically, deformed outer diameter showed a shift to the right in hypertensive arteries which remained after RDN. The axial stress - stretch curve of the hypertensive CCAs was characterized by an upward shift, while the circumferential stress - stretch curve exhibited a leftward shift when compared to control group (Fig. 1E and 1F). After RDN, both circumferential and axial stress curves restored to values similar to the control arteries.

**Figure 2:**
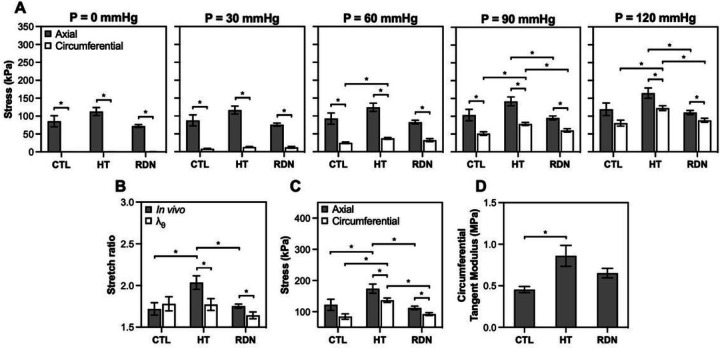
A: Comparisons of average stress in the axial and circumferential directions of the arterial wall as the pressure increases. B: Average (n=9) *in vivo* axial stretch ratio and circumferential stretch ratio at MAP. C: Average (n=9) stress in the axial and circumferential direction at MAP. D: Average (n=9) circumferential tangent modulus at MAP. Control, hypertensive and denervated group are denoted as CTL, HT and RDN, respectively. Average values are presented as mean ± SEM (*p<0.05).

Figure 2A shows the progression of axial and circumferential stresses for the three groups as a function of pressure. In all cases except control arteries at 120 mmHg, axial stress was significantly higher than the circumferential one in all groups (p < 0.05). Changes between groups started to appear at 60 mmHg. At 90 mmHg, both axial and circumferential stresses decreased after RDN, a trend that continued at 120 mmHg as well.

Figure 2B-D shows the biaxial stretch ratios and stresses in the circumferential and axial directions, and the circumferential tangent modulus at MAP. During pressurization, the axial stretch remains at *in vivo* stretch ratio while the circumferential stretch increases. In the control group, the deformation of the arterial wall reaches a close to equi-biaxial stretch state at MAP, i.e., the circumferential and axial stretches are almost the same. However, stretching appeared to be non equi-biaxial in HT and RDN groups with a significantly higher *in vivo* than circumferential stretch ratio (p < 0.05; Fig. 2B). Hypertension resulted in a significantly higher stress level from 84.6 ± 8.7 kPa to 136.6 ± 7.2 kPa (p < 0.05) in circumferential and from 122 ± 17.8 kPa to 173.8 ± 14.8 kPa (p < 0.05) in axial direction (Fig. 2C). After RDN, stresses values were restored, with a decrease to 92.1 ± 4.7 kPa (p < 0.05) and 112.2 ± 5.6 kPa (p < 0.05) in circumferential and axial direction, respectively. Tangent modulus in the circumferential direction at MAP for the three experimental groups is shown in Fig. 2D. Hypertension resulted in a significant increase of circumferential tangent modulus from 0.45 ± 0.04 MPa to 0.86 ± 0.13 MPa (p < 0.05), whereas after RDN a decrease to 0.65 ± 0.06 MPa was observed.

**Figure 3:**
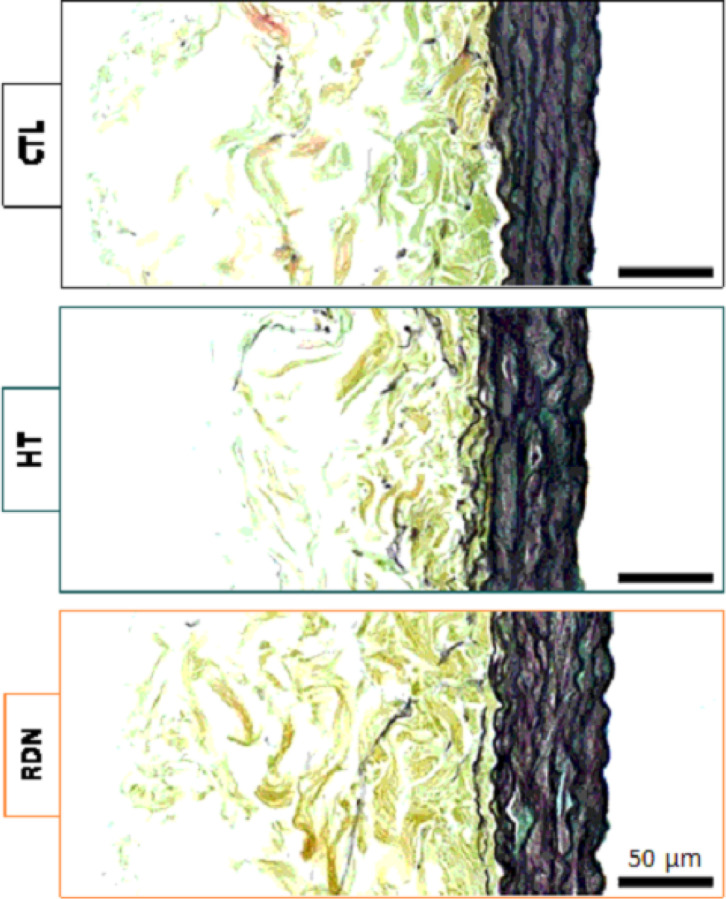
Representative histology images for the control (CTL), hypertensive (HT) and denervated (RDN) groups. Movat’s Pentachrome (MOV) stains collagen fibers yellow, elastic fibers black, smooth muscle cells red, and ground substance blue.

The structural integrity of the arteries was examined by histology and multi-photon imaging. Histology images of the CCAs are shown in Fig. 3. No significant alterations of the main arterial ECM components, elastic and collagen fibers, were observed with HT or RDN. A qualitative analysis of histology images did not show visible disruptions of the fibrous ECM components with HT and after RDN.

**Figure 4:**
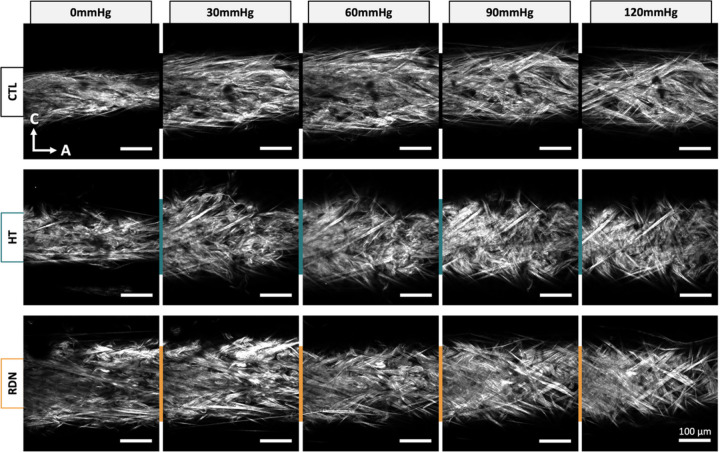
Representative multi-photon images of adventitial collagen for the control (CTL), hypertensive (HT) and denervated (RDN) group as the pressure increases. The CCAs were first stretched to *in vivo* axial stretch ratio and then pressurized between 0 and 120 mmHg. Images are 425 x 425 μm.

**Figure 5:**
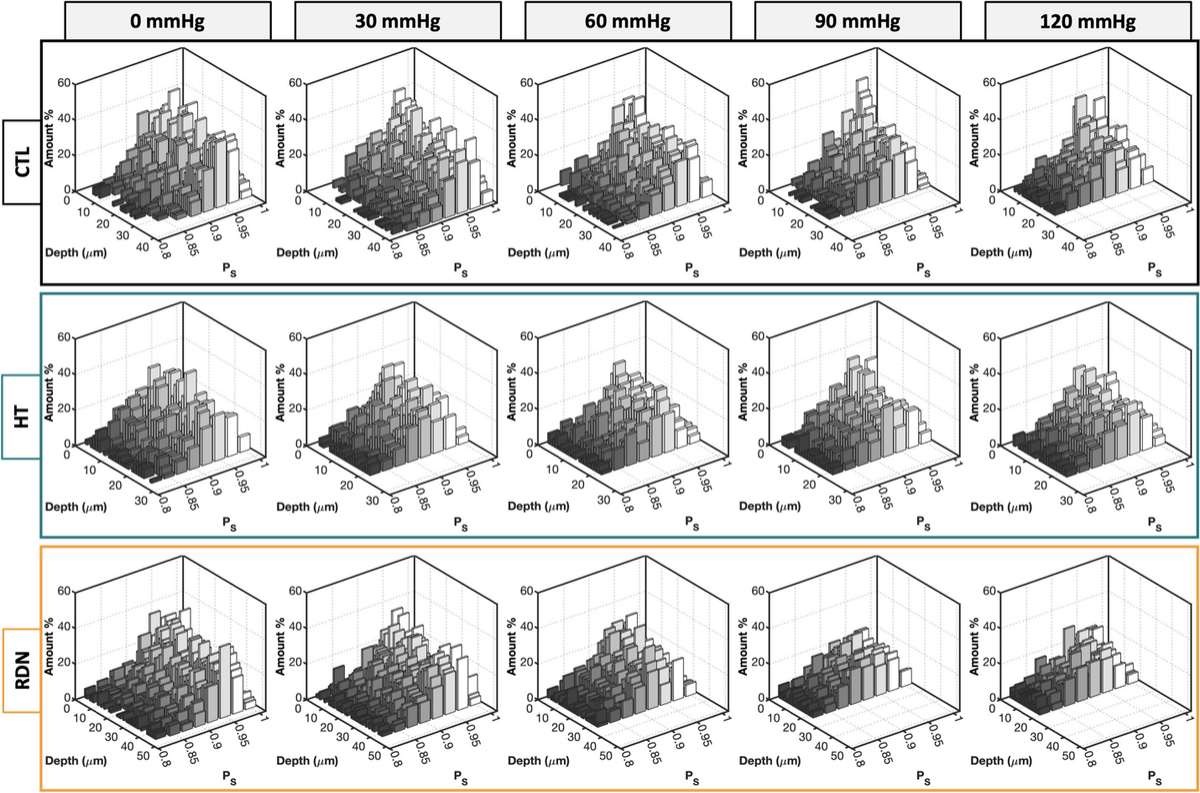
Three-dimensional (3D) histograms of straightness parameter, p_s_, as a function of pressure for the control (CTL), hypertensive (HT) and denervated (RDN) arteries in Figure 4. The 0 μm depth corresponds to the outer surface of the adventitia.

Representative multi-photon images of the adventitial collagen fibers of CCAs for the three experimental groups are given in Fig. 4. CCAs were axially stretched to their estimated *in vivo* stretch ratio and inflated at four different pressure values (0, 30, 60, 90 and 120 mmHg). Thicker collagen bundles were observed in all groups, and they were more visible at higher pressures while the fiber bundles are straightened. The corresponding 3D histograms of the straightness parameter, *P*_*s*_ of collagen fibers are given in Fig. 5 as a function of the imaging depth and pressure. In these figures, *P*_*s*_ = 1 corresponds to straight fibers whereas *P*_*s*_ < 1 corresponds to undulated fibers. Overall, a subtle straightening of the fibers with pressurization was observed. Histograms of *P*_*s*_ followed the same trend across the three groups with no visible changes within the pressure range.

**Figure 6:**
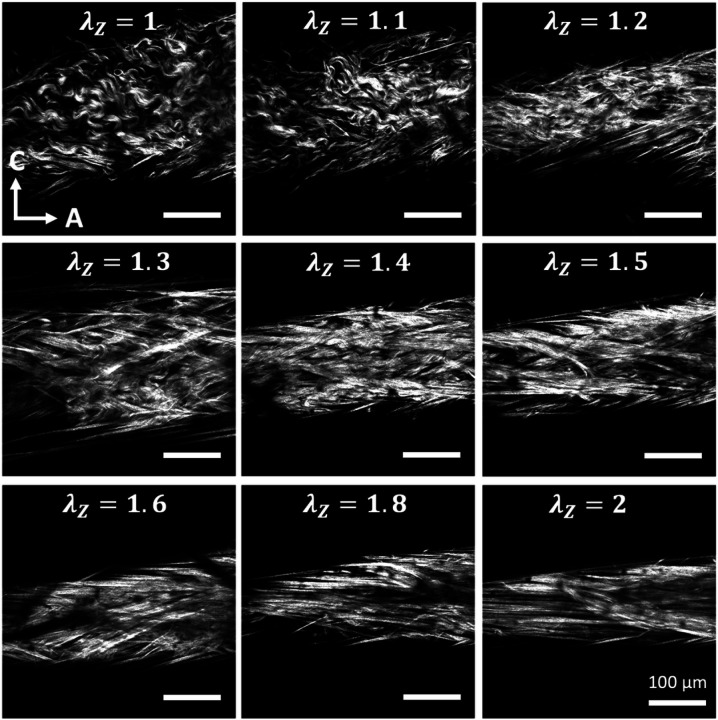
Representative multiphoton images of adventitial collagen for an artery from the control group as a function of axial stretching. Images are 425 x 425 μm.

**Figure 7:**
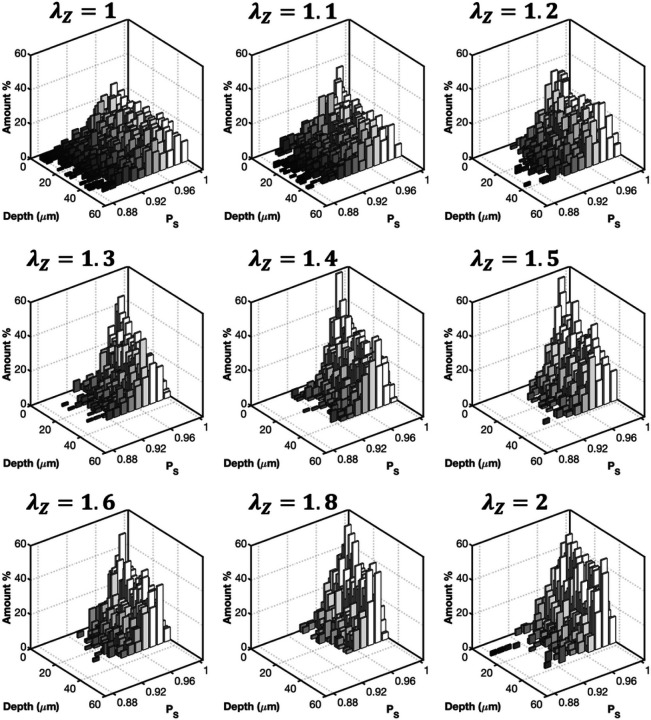
Three-dimensional (3D) histograms of straightness parameter, p_s_,as a function of axial stretching for an artery from control group in Figure 6. The 0 μm depth corresponds to the outer surface of the adventitia.

To further understand the deformation of collagen fibers, multi-photon images of CCAs were taken as a function of axial stretch at zero pressure. Representative images and the corresponding histograms of *P*_*s*_ are given in Figs. 6 and 7, respectively. Undulated collagen fibers are mainly present at lower axial stretches, while an increase of axial stretch ratio above 1.4 resulted in gradual straightening. This behavior was reflected in Fig. 7 with right-skewed *P*_*s*_ histograms which showed smaller range and higher frequency of straighter fibers (*P*_*s*_ > 0.92) for axial stretch ratios greater than 1.4. The histograms of *P*_*s*_ for the HT and RDN groups are given in the supplementary material (Figures S2 and S3).

**Figure 8:**
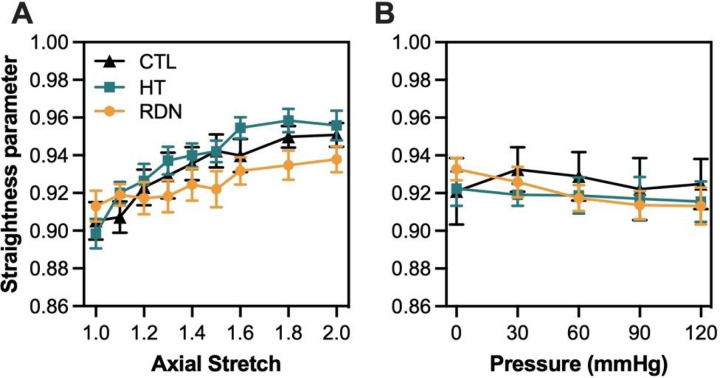
Average (mean ± SD) straightness parameter, P_s_, as a function of axial stretching (A) and pressure (B) for the control (CTL), hypertensive (HT) and denervated (RDN) group.

Since *P*_*s*_ histograms followed a skewed distribution and there were no major changes of *P*_*s*_ as a function of imaging depth (Figs. 5 and 7), the median value was extracted from every histogram. Line plots of *P*_*s*_ were generated by calculating the mean value of the median values for each collagen stack and plotted as a function of the pressure and axial stretch in Fig. 8A and 8B, respectively. As the arteries were stretched in the axial direction, collagen fibers showed an increasing trend of straightening which plateaued above 1.8 stretch (Fig. 8A). On the other hand, when arteries were fixed at their *in vivo* stretch ratio *P*_*s*_ remain almost constant with pressurization with no significant variations, regardless of the experimental group (Fig. 8B).

**Figure 9:**
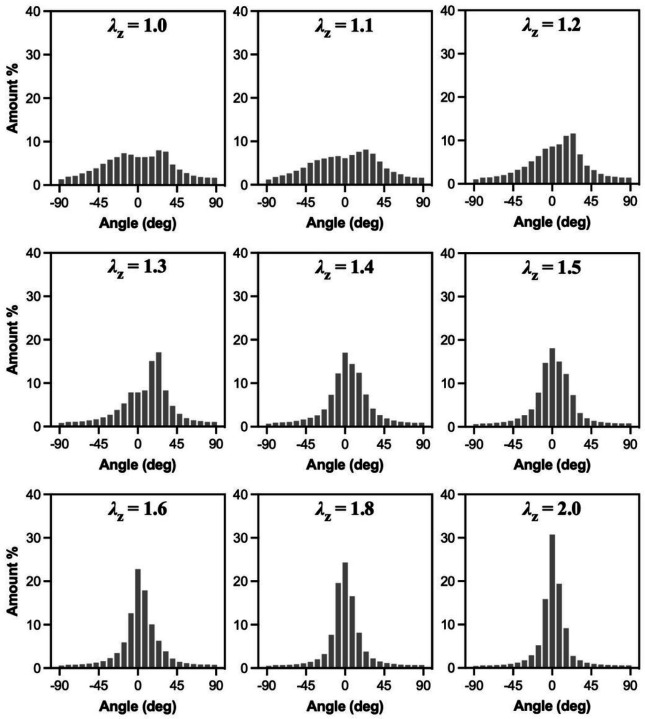
Histograms of collagen fibers orientation for the control group as a function of axial stretching. Here 0° and ±90° corresponding to the axial and circumferential direction, respectively.

**Figure 10:**
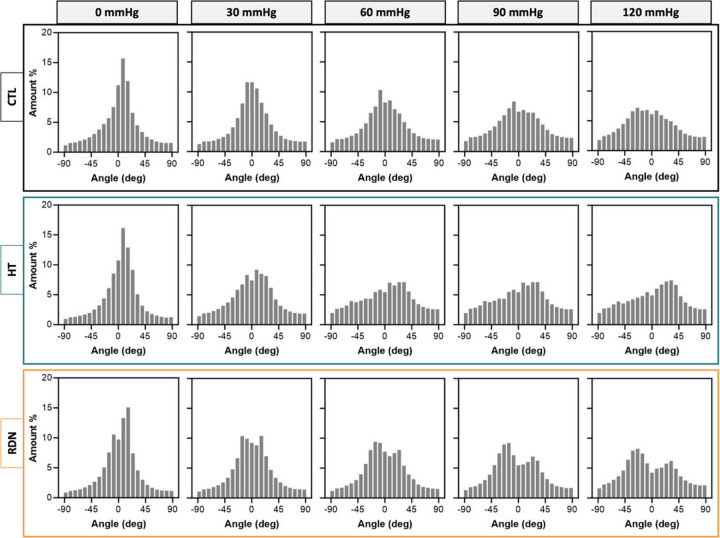
Histograms of collagen fibers orientation for the control (CTL), hypertensive (HT) and denervated (RDN) group as the pressure increase from 0 to 120 mmHg. Here 0° and ±90° corresponding to the axial and circumferential direction, respectively.

Figures 9 and 10 show collagen fibers orientation as a function axial stretch ratio and pressure, respectively. Since there were no differences in the collagen fiber orientation with axial stretch ratio between the three groups, only histograms for the control arteries are shown in Fig. 9. The histograms of collagen orientation for the HT and RDN groups are given in the supplementary material (Figure S4 and S5, for HT and RDN, respectively). The orientation of collagen fibers seemed to be varied mostly ±45° from the axial direction at low stretch ratios and aligned towards the axial direction with further axial stretching (Fig. 9). On the other hand, pressurization of the arteries resulted in a shifting of orientation from a bell-shaped histogram around 0° (axial direction) towards a multimodal histogram with multiples peaks which ranged from − 45° to 45° (Fig. 10). For the denervated arteries, orientation started as a bimodal histogram with two peaks around axial direction, while those two peaks became more distinct and apart at higher pressures.

**Figure 11:**
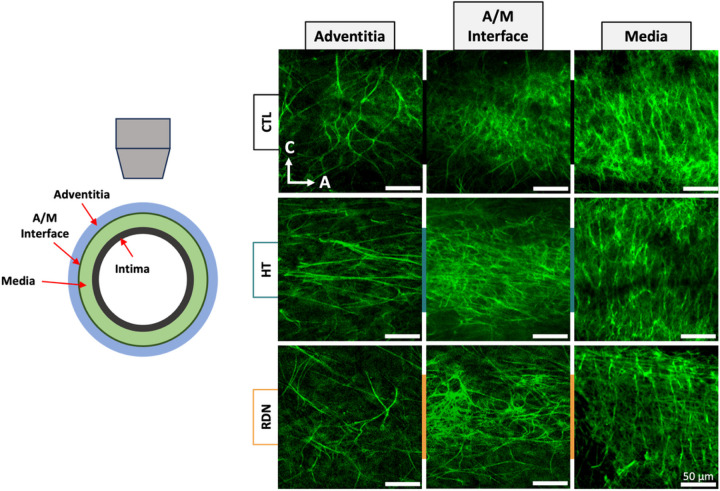
Representative multiphoton images of elastic fibers at 90 mmHg for the control (CTL), hypertensive (HT) and denervated (RDN) experimental group at three different imaging depths representing the adventitia (left), adventitia media (A/M) interface (middle) and media (right). Images are 203 x 203 μm.

**Figure 12:**
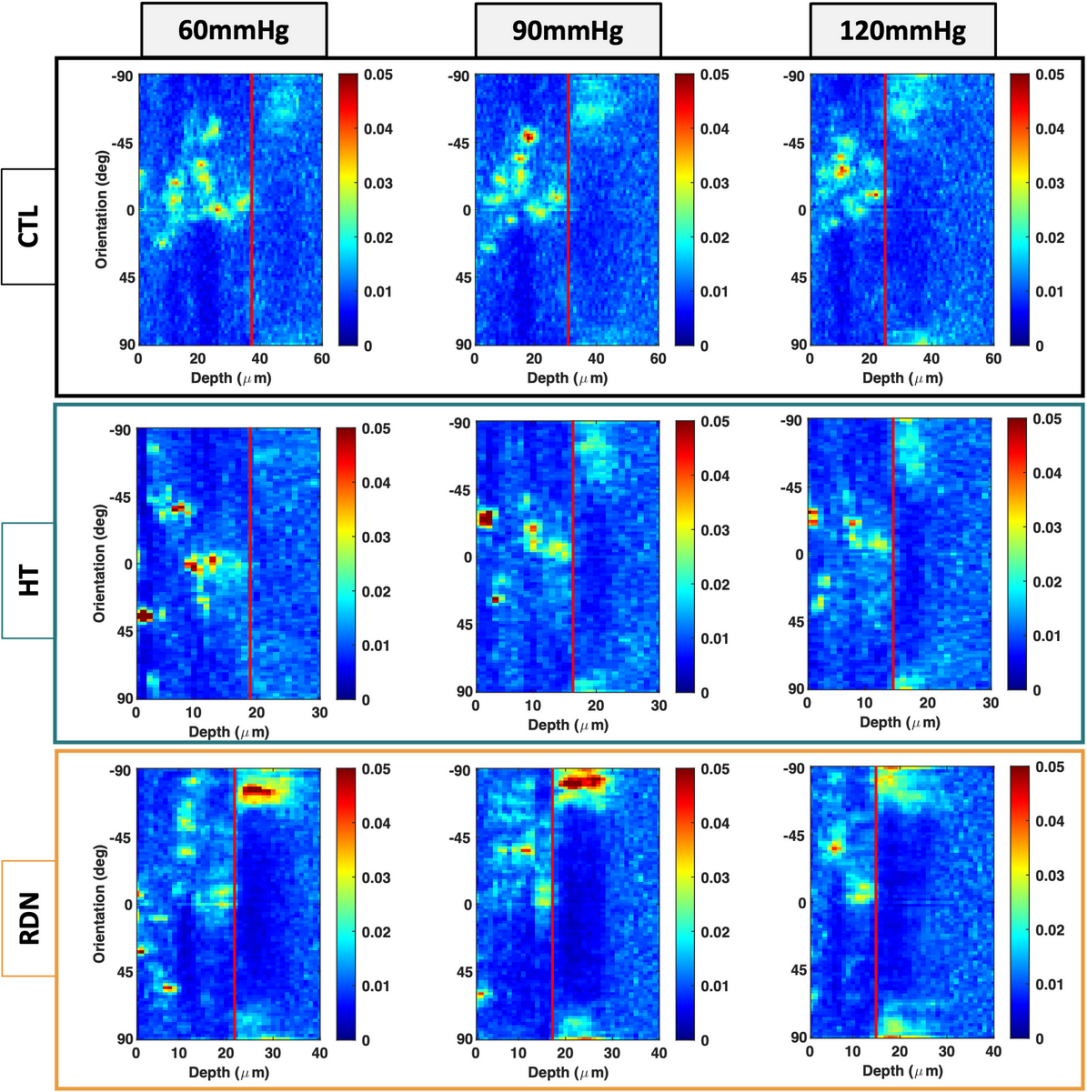
Representative orientation maps of the elastic fibers at 60, 90 and 120 mmHg as a function of the imaging depth for the control (CTL), hypertensive (HT) and denervated (RDN) group. Here 0° and ± 90° correspond to the axial and circumferential direction, respectively, and 0 μm depth corresponds to the outer surface of the adventitia. Scale bars represent the amount (%) of elastic fibers oriented in each direction. In all figures adventitia-media interface was visually identified and marked with a red line.

Figure 11 shows representative multi-photon images of the elastic fibers under a pressure of 90 mmHg. The three columns show images at three different transmural sites: adventitia (left column), interface between adventitia and media (denoted as A/M interface; middle column), and outer media (right column). Sparse elastic fibers were present in adventitia in all groups, with a disperse orientation. At the interface, elastic fibers started to form a denser network. In the outer layers of media, elastic fibers showed a preferred orientation towards the circumferential direction. Representative orientation of the elastic fibers is given in Fig. 12 as a function of the imaging depth and pressure (60 mmHg, 90 mmHg and 120 mmHg). Adventitial elastic fibers in CCA from the control group oriented around the axial direction throughout the applied pressure. Elastic fibers of the hypertensive CCA were distributed at ± 45° for the first adventitial layers and then appeared to change their orientation to the axial direction. After RDN, adventitial elastic fibers were randomly distributed from 45° to −90°. An abrupt change of the elastic fibers orientation from axial to circumferential was observed in all samples (red line). After careful examination of this transitional area in the multi-photon stack of images, it was identified as the interface between adventitia and media.

**Figure 13:**
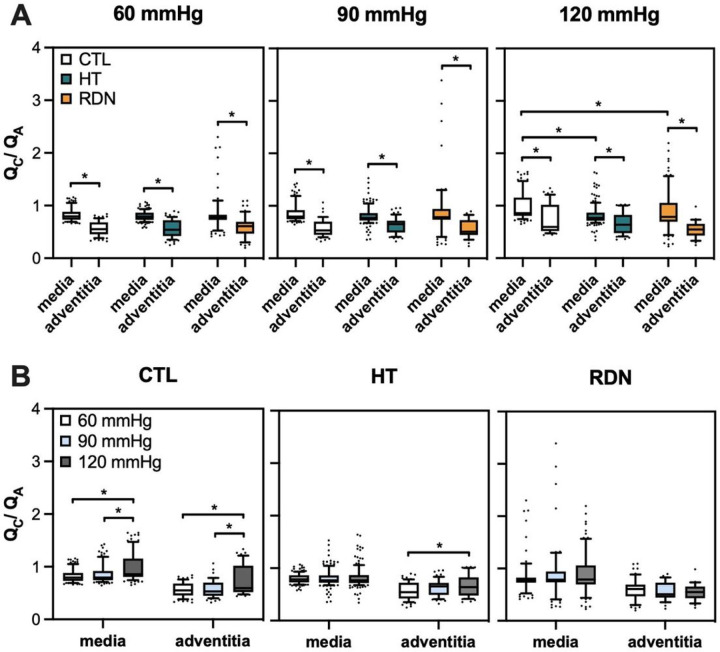
A: Average ratio (mean ± SD) of circumferential-to-axial oriented elastic fibers in media and adventitia at 60, 90 and 120 mmHg. B: Average ratio (mean ± SD) of circumferential-to-axial oriented elastic fibers for the three experimental groups CTL, HT, and RDN (*p<0.05).

To further understand the differences in elastic fiber distributions in the arterial wall, the ratio of circumferential-to-axial (*Q*_*C*_ /*Q*_*A*_) oriented elastic fibers was obtained and given in Fig. 13. The top row shows the *Q*_*C*_ /*Q*_*A*_ for three different pressures (60, 90 and 120 mmHg) and the bottom row shows the *Q*_*C*_ /*Q*_*A*_ for the three groups. Significance bars between media and adventitia are shown only in the top row figures. As it was noticed before, the elastic fibers show a preferred orientation towards the axial direction in adventitia with a *Q*_*C*_ /*Q*_*A*_ < 1, and change to preferred circumferential distribution in the outer media. At the physiological pressure of 120 mmHg, *Q*_*C*_ /*Q*_*A*_ in media decreased significantly from 0.99 ± 0.28 to 0.82 ± 0.2 and 0.89 ± 0.39 for the HT and RDN when compared to control group (p < 0.05) indicating an orientation shifting towards the axial direction. Differences of the *Q*_*C*_ /*Q*_*A*_ were observed as a function of pressure for the control and HT groups. Specifically, *Q*_*C*_ /*Q*_*A*_ ratio increased significantly in the media for the control group from 0.83 ± 0.12 at 60 mmHg and 0.87 ± 0.2 at 90 mmHg to 0.99 ± 0.28 at 120 mmHg, and in the adventitia from 0.56 ± 0.14 at 60 mmHg and 0.57 ± 0.16 at 90 mmHg to 0.76 ± 0.28 at 120 mmHg (p < 0.05; Fig. 13B). For the HT group, *Q*_*C*_ /*Q*_*A*_ ratio increased significantly only in the adventitia from 0.57 ± 0.17 at 60 mmHg to 0.66 ± 0.2 at 120 mmHg (p < 0.05; Fig. 13B).

## Discussion

Sympathetic nervous system and the kidney play an important role in regulating blood pressure. In this study, we investigated the biomechanical response of CCAs in hypertension and after RDN using a rat model. Our results suggest a sustained elevated blood pressure resulted in arterial remodeling manifested by significantly increased circumferential and axial stresses in carotid arteries. RDN of 8 months old rats effectively reversed the biomechanical response of carotid arteries with no obvious structural remodeling in the ECM. Mean blood pressure and biaxial stresses were restored to normotensive values, with significant changes in stresses, *in vivo* stretch ratio, and axial force. Microstructural imaging and quantification revealed intact ECM in the denervated CCAs.

In the present study, mechanical properties of carotid arteries from the hypertensive group were impacted with increased biaxial stresses and tangent modulus (Fig. 2C and 2D). The positive effect of RDN was seen not only on hemodynamics as a decrease in MAP, but also on restoration of arterial biomechanical response. After RDN, passive wall biaxial stresses and arterial stiffness were restored to similar ranges for the 3-month group (Fig. 2C and 2D). Clinical studies have shown a decrease in blood pressure, as well as reverse arterial stiffening following RDN measured by pulse wave velocity (PWV) (33–35). In these studies, PWV measurement at 1, 3, 6 and 12 months after RDN showed a significant decrease that started one day after renal denervation, while the PWV reduction sustained after a 12-month follow-up (33, 35). Given that the early changes in PWV after RDN were independent of blood pressure, the authors suggested that the decrease in PWV might be associated with a modification of sympathetic tone (33).

One interesting finding in this study is the effect of hypertension and RDN in the axial biomechanics. Hypertension resulted in increased *in vivo* stretch ratio and axial force, while RDN restored the values of both metrics (Figs. 1B and 1C). Remodeling in axial direction of an elastic artery is one of the first structural changes detected during the early stages of postnatal growth and development, manifested by an increased axial stretching due to a body length growth, and a nonuniform axial wall thickening (36, 37). Arteries remain prestretched in the axial direction with maturity, and exhibit minimal axial deformation during cardiac cycle, which is energetically favorable and facilitates the propagation of pulse pressure (38, 39). Organ culture studies reported that arteries adapted to their biomechanical environment by increasing their length when subjected to sustained increased axial stretch or loading (40–42). Morphological changes in axial configuration were noticed with aging as well as hypertension, and include an elongation of arterial segments due to a deposition of stressed collagen which resulted in a decreased in *vivo* stretch ratio; however, studies on axial force resulted in contradictory findings (43–48). Nevertheless, limited information is available on axial remodeling and biomechanical response of elastic arteries due to the lack of methods to accurately measure and obtain *in vivo* values of axial prestretch and stress (48). The arteries are unlikely to grow in the axial direction from the 3- to 8 month’s groups; however, the axial force needed to maintain arteries in their *in vivo* length increased (Fig. 2C). Another interesting finding of this study is the difference in biaxial stress/stretch state of the arteries under physiological loading among the three groups. At MAP, our results indicate that the 3-month control group were subjected to a nearly equibiaxal stretching, i.e., the *in vivo* axial stretch and the circumferential stretch are the same, while the 8-month hypertensive and RDN arteries were subjected to a non-equibiaxial stretching with a significantly higher axial than circumferential stress/stretch (Fig. 2B). The biaxial stress state will likely play an important role in regulating cellular behavior and ECM structural organization in physiology and diseases. Understanding the altered biaxial stress/stretch state in the 8-months groups may potentially help us to explain the biomechanical changes in the axial direction.

While thickening of the arterial wall in hypertension is often seen as a compensatory mechanism to restore the increased wall stresses towards normal, homeostatic values (49), in our study, the sustained elevated blood pressure led to an enlargement of the arteries but with no significant changes in total thickness (Figure S1). The lack of significant changes in wall thickness in the present study (Figure S1D) could be explained by the time course of arterial remodeling. Previous studies have shown a temporal progression of changes in matrix synthesis and mechanical properties in hypertension. Specifically, thickness of the arterial wall showed an increasing trend which peaked 2 weeks after inducing hypertension and started decreasing at 42 days, while stiffness increased during the first week and reached a plateau after (45, 50). These findings correlate well with an immunohistochemical analysis of porcine aorta during hypertension which showed increased collagen production at 2 weeks of hypertension; however, collagen degradation resulted in a decrease in collagen content at 6 and 8 weeks (51). It should be noted that in these studies, hypertension was induced by constricted the aorta or renal arteries, and changes were monitored for a specific time window after hypertension, ranging from 0 days to 16 weeks, whereas in our study we used 3 and 8 months old hypertensive rats with no information about the exact starting time point for hypertension.

The structural organization of the fibrous ECM components, i.e. collagen and elastic fibers, has long been shown to play an important role in determining the mechanical behavior of elastic arteries. Studies investigating the relationship between the arterial microstructure and its mechanical response showed that the arterial wall exhibits a nonlinear and anisotropic behavior under loading with the elastic fibers being the primary source of elasticity, and collagen fiber waviness and gradual recruitment contributing to nonlinearity (52–55). Previous studies have further investigated the layer-specific microstructure and its correlation to arterial mechanical properties (31, 56, 57). These studies showed sequential engagement of elastic and collagen fibers under biaxial loading. In our study, structural information (waviness and orientation) of collagen and elastic fibers in adventitial and outer media was extracted during biaxial inflation and extension. Quantification of adventitial collagen fibers microstructure showed no change in straightness parameter during pressurization from 0 to 120 mmHg while arteries were deformed to their *in vivo* stretch ratio (Fig. 8B); however, incremental stretching in the axial direction revealed a gradual straightening of collagen fibers which reached a plateau for axial ratios greater than 1.6 (Fig. 8A). Given that the *in vivo* stretch ratio in the present study is higher than 1.7 in all groups, our results show that the unfolding of the adventitial collagen fibers takes place during the axial stretching of the arteries, and thus possibly contribute to macroscopic deformation in the axial direction. Interestingly, pressurization resulted in a reorientation of adventitial collagen fibers from the axial direction to more dispersed or two fiber families in the diagonal direction (± 45°; Fig. 10). These findings add to the existing knowledge about collagen orientation under loading and indicate that collagen fibers straighten during axial stretching, whereas reorientation may be the main microstructural mechanism that takes place during pressurization.

Quantification of multi-photon images showed no significant changes in straightness parameter and orientation between control and hypertensive arteries within a physiological pressure range (Figs. 8B and 10). Despite that the microstructural quantifications from this study cannot be directly related to the increase in stresses and tangent modulus in the hypertensive group, compromised quality of the existing ECM components remains to be a possibility. Murtada et al. (58) have shown that elastic fibers in mice thoracic aorta remained intact with no increase in the amount of elastin after angiotensin II induced hypertension. However, elastic fibers were less effective and appeared to have a reduced ability to store elastic energy. Besides elastic fibers, alterations in collagen subtypes proportions were also associated with arterial stiffness, regardless of changes in collagen amount. An altered collagen type-I turnover was found to be correlated with arterial stiffness in hypertensive subjects with an increased collagen type-I degradation (59). In addition, arterial stiffness was correlated with an upregulation of collagen type-V in arteries from hypertensive rats while the amount of collagen type-I decreased (60). The effect of RDN on ECM remodeling has been investigated in a few studies but its connection to arterial stiffness remains largely unknown. Studies performed histology analysis of arterial samples after RDN and reported a decreased expression of Angiotensin II receptor (61), reduced vascular fibrosis (62) and inhibition of NOX activity which indicated an attenuated oxidative stress (63), all of which have been known to be associated with arterial stiffness (64–66). Our multi-photon and histology images of collagen and elastic fibers suggested that RDN resulted in no structural changes, leaving arterial tissues seemed intact at the microscopic level (Figs. 3, 4 and 11). The reversed biomechanical response after RDN (stresses and stiffness) were likely related to alterations in ECM composition of arterial wall resulted from combined neurological and hemodynamical modifications. Future studies focusing on fiber mechanics and biochemical compositional analysis of ECM constituents may yield insightful findings that advance our understandings on vascular mechanics and microstructural composition.

### Limitations

Multi-photon microscopy has emerged as a promising imaging technique since it requires minimal tissue preparation, however the imaging depth in intact arterial tissue is restricted to ~ 50–60 µm due to light scattering (30, 31, 67). Given that the adventitia thickness is usually 1/3 of the total thickness of the arterial wall (30, 68, 69), we were able to image the adventitia and only the outer layers of media. Generalizations of our results to the entire medial layer should be made cautiously. In addition, quantification of collagen and elastic fiber structure (waviness and orientation) was performed in the two-dimensional imaging plane. However, ECM fibers usually cross planes and thus a three dimensional (3D) reconstruction of the collagen fibers is more representative than the (2D) analysis that was followed in this study (70). The MAP in all groups was higher than 120 mmHg, which was the highest applied pressure during imaging. However, since arteries already reach stiffening at 120 mmHg, we do not expect much noticeable structural changes at higher pressures. Moreover, vascular smooth muscle cells (VSMC) play an important role in regulating vascular properties during physiological and pathophysiological conditions. Specifically in hypertension, VSMC differentiate their phenotype from a contractile to a proliferative and synthetic, which affect the arterial response to loading (71). However, the main objective of this study was to investigate the passive mechanical properties and microstructure of the arterial wall in hypertension and RDN, hence we did not take into consideration the contribution of VSMC. While it has been reported that the renal nerves fully reinnervate following renal denervation over a 12-week period (72) we are confident in the efficacy of our renal denervation procedure that reduced renal norepinephrine content to less than 10% of that seen in control animals. As such we do not believe that is a confounding effect of renal nerve reinnervation in the current studies.

## Conclusions

Renal denervation has emerged as a therapeutic potential in cases of resistant hypertension with the interplay between sympathetic nervous system and kidney in regulating blood pressure. Our study showed that hypertension in 8 months old rats resulted in altered arterial mechanical response with increased stresses in both axial and circumferential direction, as well as increased circumferential tangent modulus. RDN attenuated mean arterial pressure in 8 months old hypertensive rats. In addition, it effectively restored the biomechanical response of the CCAs to normal values with a reverse of axial and circumferential stresses to values similar to control arteries (3 months old). Microstructural analysis of the arterial wall via multi-photon microscopy revealed that the fibrous components of the ECM remained intact after RDN. Our results indicated an impact of hypertension and renal denervation on axial biomechanical properties, i.e. axial force and *in vivo* axial stretch ratio. Regardless of hypertension and renal denervation, our study also provides information on the ECM microstructural changes during loading. Undulated collagen fibers were present in load-free tissues and their straightening started during axial stretching up to their estimated *in vivo* axial stretch ratio. Interestingly, collagen fiber reorientation was the main microstructural mechanism during pressurization. Our study provides interesting initial findings on the effect of effect of RDN on large elastic arteries mechanical response and microstructure. Future studies are needed to fully reveal the complex interplay between sympathetic nervous system and blood pressure regulation and as well as the effectiveness of RDN.

## Supplementary Material

Supplement 1

## References

[1] Humphrey JD, Dufresne ER, Schwartz MA. Mechanotransduction and extracellular matrix homeostasis. Nature Reviews Molecular Cell Biology 15: 802–812, 2014. doi: 10.1038/nrm3896. 25355505 PMC4513363

[2] Sherratt MJ. Tissue elasticity and the ageing elastic fibre. AGE 31: 305–325, 2009. doi: 10.1007/s11357-009-9103-6. 19588272 PMC2813052

[3] Sun Z. Aging, arterial stiffness, and hypertension. Hypertension 65: 252–256, 2015. doi: 10.1161/HYPERTENSIONAHA.114.03617. 25368028 PMC4288978

[4] Humphrey JD, Harrison DG, Figueroa CA, Lacolley P, Laurent S. Central Artery Stiffness in Hypertension and Aging. Circulation Research 118: 379–381, 2016. doi: 10.1161/CIRCRESAHA.115.307722. 26846637 PMC4745997

[5] Laurent S, Boutouyrie P. The Structural Factor of Hypertension. Circulation Research 116: 1007–1021, 2015. doi: 10.1161/CIRCRESAHA.116.303596. 25767286

[6] Safar ME, Asmar R, Benetos A, Blacher J, Boutouyrie P, Lacolley P, Laurent S, London G, Pannier B, Protogerou A, Regnault V, null null. Interaction Between Hypertension and Arterial Stiffness. Hypertension 72: 796–805, 2018. doi: 10.1161/HYPERTENSIONAHA.118.11212. 30354723

[7] Humphrey JD. Mechanisms of Arterial Remodeling in Hypertension. Hypertension 52: 195–200, 2008. doi: 10.1161/HYPERTENSIONAHA.107.103440. 18541735 PMC2753501

[8] Intengan HD, Schiffrin EL. Vascular Remodeling in Hypertension. Hypertension 38: 581–587, 2001. doi: 10.1161/hy09t1.096249. 11566935

[9] Briones AM, Arribas SM, Salaices M. Role of extracellular matrix in vascular remodeling of hypertension [Online]. Current Opinion in Nephrology and Hypertension 19, 2010. https:/journals.lww.com/co-nephrolhypertens/Fulltext/2010/03000/Role_of_extracellular_matrix_in_vascular.12.aspx. 10.1097/MNH.0b013e328335eec9 20040870

[10] Weisbrod RM, Shiang T, Al Sayah L, Fry JL, Bajpai S, Reinhart-King CA, Lob HE, Santhanam L, Mitchell G, Cohen RA, Seta F. Arterial Stiffening Precedes Systolic Hypertension in Diet-Induced Obesity. Hypertension 62: 1105–1110, 2013. doi: 10.1161/HYPERTENSIONAHA.113.01744. 24060894 PMC3951434

[11] Bussy C, Boutouyrie P, Lacolley P, Challande P, Laurent S. Intrinsic Stiffness of the Carotid Arterial Wall Material in Essential Hypertensives. Hypertension 35: 1049–1054, 2000. doi: 10.1161/01.HYP.35.5.1049. 10818063

[12] Zhang Y, Lacolley P, Protogerou AD, Safar ME. Arterial Stiffness in Hypertension and Function of Large Arteries. American Journal of Hypertension 33: 291–296, 2020. doi: 10.1093/ajh/hpz193. 32060496

[13] Eberth JF, Cardamone L, Humphrey JD. Evolving biaxial mechanical properties of mouse carotid arteries in hypertension. Journal of Biomechanics 44: 2532–2537, 2011. doi: 10.1016/j.jbiomech.2011.07.018. 21851943 PMC3169381

[14] Iliescu R, Lohmeier TE, Tudorancea I, Laffin L, Bakris GL. Renal denervation for the treatment of resistant hypertension: review and clinical perspective. Am J Physiol Renal Physiol 309: F583–F594, 2015. doi: 10.1152/ajprenal.00246.2015. 26224718 PMC4593817

[15] Frame AA, Carmichael CY, Wainford RD. Renal Afferents. Current Hypertension Reports 18: 69, 2016. doi: 10.1007/s11906-016-0676-z. 27595156 PMC5011151

[16] Kannan A, Medina RI, Nagajothi N, Balamuthusamy S. Renal sympathetic nervous system and the effects of denervation on renal arteries. World J Cardiol 6: 814–823, 2014. doi: 10.4330/wjc.v6.i8.814. 25228960 PMC4163710

[17] Katholi RE, Rocha-Singh KJ. The Role of Renal Sympathetic Nerves in Hypertension: Has Percutaneous Renal Denervation Refocused Attention on Their Clinical Significance? Progress in Cardiovascular Diseases 52: 243–248, 2009. doi: 10.1016/j.pcad.2009.09.003. 19917336

[18] Fengler K, Rommel K-P, Blazek S, Besler C, Hartung P, von Roeder M, Petzold M, Winkler S, Höllriegel R, Desch S, Thiele H, Lurz P. A Three-Arm Randomized Trial of Different Renal Denervation Devices and Techniques in Patients With Resistant Hypertension (RADIOSOUND-HTN). Circulation 139: 590–600, 2019. doi: 10.1161/CIRCULATIONAHA.118.037654. 30586691

[19] Walsh KR, Kuwabara JT, Shim JW, Wainford RD. Norepinephrine-evoked salt-sensitive hypertension requires impaired renal sodium chloride cotransporter activity in Sprague-Dawley rats. American Journal of Physiology-Regulatory, Integrative and Comparative Physiology 310: R115–R124, 2016. doi: 10.1152/ajpregu.00514.2014. 26608659 PMC4796647

[20] Frame AA, Puleo F, Kim K, Walsh KR, Faudoa E, Hoover RS, Wainford RD. Sympathetic regulation of NCC in norepinephrine-evoked salt-sensitive hypertension in Sprague-Dawley rats. American Journal of Physiology-Renal Physiology 317: F1623–F1636, 2019. doi: 10.1152/ajprenal.00264.2019. 31608673 PMC6960786

[21] Kapusta DR, Pascale CL, Kuwabara JT, Wainford RD. Central Nervous System Gαi2-Subunit Proteins Maintain Salt Resistance via a Renal Nerve–Dependent Sympathoinhibitory Pathway. Hypertension 61: 368–375, 2013. doi: 10.1161/HYPERTENSIONAHA.111.00014. 23213191 PMC3562703

[22] Wainford RD, Kapusta DR. Chronic high-NaCl intake prolongs the cardiorenal responses to central N/OFQ and produces regional changes in the endogenous brain NOP receptor system. American Journal of Physiology-Regulatory, Integrative and Comparative Physiology 296: R280–R288, 2009. doi: 10.1152/ajpregu.00096.2008. 18987291 PMC2643980

[23] Wainford R, Kapusta D. Functional selectivity of central Gα-subunit proteins in mediating the cardiovascular and renal excretory responses evoked by central α2-adrenoceptor activation in vivo. British Journal of Pharmacology 166: 210–220, 2012. doi: 10.1111/j.1476-5381.2011.01662.x. 21895632 PMC3415649

[24] Wainford RD, Pascale CL, Kuwabara JT. Brain Gαi2-subunit protein-gated pathways are required to mediate the centrally evoked sympathoinhibitory mechanisms activated to maintain sodium homeostasis [Online]. Journal of Hypertension 31, 2013. https:/journals.lww.com/jhypertension/Fulltext/2013/04000/Brain_G_i2_subunit_protein_gated_pathways_are.17.aspx. 10.1097/HJH.0b013e32835ebd54 23391983

[25] Wainford RD, Carmichael CY, Pascale CL, Kuwabara JT. Gαi2-Protein–Mediated Signal Transduction. Hypertension 65: 178–186, 2015. doi: 10.1161/HYPERTENSIONAHA.114.04463. 25312437 PMC4268057

[26] Carmichael CY, Kuwabara JT, Pascale CL, Moreira JD, Mahne SE, Kapusta DR, Rosene DL, Williams JS, Cunningham JT, Wainford RD. Hypothalamic Paraventricular Nucleus Gαi2 (Guanine Nucleotide–Binding Protein Alpha Inhibiting Activity Polypeptide 2) Protein–Mediated Neural Control of the Kidney and the Salt Sensitivity of Blood Pressure. Hypertension 75: 1002–1011, 2020. doi: 10.1161/HYPERTENSIONAHA.119.13777. 32148128 PMC7329357

[27] Puleo F, Kim K, Frame AA, Walsh KR, Ferdaus MZ, Moreira JD, Comsti E, Faudoa E, Nist KM, Abkin E, Wainford RD. Sympathetic Regulation of the NCC (Sodium Chloride Cotransporter) in Dahl Salt–Sensitive Hypertension. Hypertension 76: 1461–1469, 2020. doi: 10.1161/HYPERTENSIONAHA.120.15928. 32981364 PMC7727920

[28] Gkousioudi A, Yu X, Ferruzzi J, Qian J, Wainford RD, Seta F, Zhang Y. Biomechanical Properties of Mouse Carotid Arteries With Diet-Induced Metabolic Syndrome and Aging [Online]. Frontiers in Bioengineering and Biotechnology 10, 2022. 10.3389/fbioe.2022.862996. PMC8980683 35392404

[29] Ferruzzi J, Bersi MR, Humphrey JD. Biomechanical phenotyping of central arteries in health and disease: advantages of and methods for murine models. Ann Biomed Eng 41: 1311–1330, 2013. doi: 10.1007/s10439-013-0799-1. 23549898 PMC3918742

[30] Yu X, Turcotte R, Seta F, Zhang Y. Micromechanics of elastic lamellae: unravelling the role of structural inhomogeneity in multi-scale arterial mechanics. Journal of The Royal Society Interface 15: 20180492, 2018. doi: 10.1098/rsif.2018.0492. 30333250 PMC6228495

[31] Chow M-J, Turcotte R, Lin CP, Zhang Y. Arterial Extracellular Matrix: A Mechanobiological Study of the Contributions and Interactions of Elastin and Collagen. Biophysical Journal 106: 2684–2692, 2014. doi: 10.1016/j.bpj.2014.05.014. 24940786 PMC4070071

[32] Bredfeldt Jeremy S., Liu Yuming, Pehlke Carolyn A., Conklin Matthew W., Szulczewski Joseph M., Inman David R., Keely Patricia J., Nowak Robert D., Mackie Thomas R., Eliceiri Kevin W.. Computational segmentation of collagen fibers from second-harmonic generation images of breast cancer. Journal of Biomedical Optics 19: 016007, 2014. doi: 10.1117/1.JBO.19.1.016007. 24407500 PMC3886580

[33] Berukstis A, Navickas R, Neverauskaite-Piliponiene G, Ryliskyte L, Misiura J, Vajauskas D, Misonis N, Laucevicius A. Arterial Destiffening Starts Early after Renal Artery Denervation. International Journal of Hypertension 2019: 3845690, 2019. doi: 10.1155/2019/3845690. 30941209 PMC6421020

[34] Ott C, Franzen KF, Graf T, Weil J, Schmieder RE, Reppel M, Mortensen K. Renal denervation improves 24-hour central and peripheral blood pressures, arterial stiffness, and peripheral resistance. The Journal of Clinical Hypertension 20: 366–372, 2018. 29370474 10.1111/jch.13193 PMC8030791

[35] Brandt Mathias C., Reda Sara, Mahfoud Felix, Lenski Matthias, Böhm Michael, Hoppe Uta C. Effects of Renal Sympathetic Denervation on Arterial Stiffness and Central Hemodynamics in Patients With Resistant Hypertension. Journal of the American College of Cardiology 60: 1956–1965, 2012. doi: 10.1016/j.jacc.2012.08.959. 23062529

[36] Dobrin P, Canfield T, Sinha S. Development of longitudina retraction of carotid arteries in neonatal dogs. Experientia 31: 1295–1296, 1975. doi: 10.1007/BF01945788. 1204778

[37] Huang Y, Guo X, Kassab GS. Axial nonuniformity of geometric and mechanical properties of mouse aorta is increased during postnatal growth. American Journal of Physiology-Heart and Circulatory Physiology 290: H657–H664, 2006. doi: 10.1152/ajpheart.00803.2005. 16172154

[38] Horný L, Netušil M, Voňavková T. Axial prestretch and circumferential distensibility in biomechanics of abdominal aorta. Biomechanics and Modeling in Mechanobiology 13: 783–799, 2014. doi: 10.1007/s10237-013-0534-8. 24136338

[39] Cardamone L, Valentín A, Eberth JF, Humphrey JD. Origin of axial prestretch and residual stress in arteries. Biomechanics and Modeling in Mechanobiology 8: 431, 2009. doi: 10.1007/s10237-008-0146-x. 19123012 PMC2891240

[40] Davis NP, Han H-C, Wayman B, Vito R. Sustained axial loading lengthens arteries in organ culture. Annals of biomedical engineering 33: 867–877, 2005. 16060526 10.1007/s10439-005-3488-x

[41] Lehman RM, Owens GK, Kassell NF, Hongo K. Mechanism of enlargement of major cerebral collateral arteries in rabbits. Stroke 22: 499–504, 1991. doi: 10.1161/01.STR.22.4.499. 2024279

[42] Sho E, Nanjo H, Sho M, Kobayashi M, Komatsu M, Kawamura K, Xu C, Zarins CK, Masuda H. Arterial enlargement, tortuosity, and intimal thickening in response to sequential exposure to high and low wall shear stress. Journal of Vascular Surgery 39: 601–612, 2004. doi: 10.1016/j.jvs.2003.10.058. 14981455

[43] Vaishnav RN, Vossoughi J, Patel DJ, Cothran LN, Coleman BR, Ison-Franklin EL. Effect of Hypertension on Elasticity and Geometry of Aortic Tissue From Dogs. Journal of Biomechanical Engineering 112: 70–74, 1990. doi: 10.1115/1.2891128. 2308306

[44] Bersi MR, Bellini C, Wu J, Montaniel KRC, Harrison DG, Humphrey JD. Excessive Adventitial Remodeling Leads to Early Aortic Maladaptation in Angiotensin-Induced Hypertension. Hypertension 67: 890–896, 2016. doi: 10.1161/HYPERTENSIONAHA.115.06262. 27001298 PMC4833633

[45] Eberth JF, Popovic N, Gresham VC, Wilson E, Humphrey JD. Time course of carotid artery growth and remodeling in response to altered pulsatility. American Journal of Physiology-Heart and Circulatory Physiology 299: H1875–H1883, 2010. doi: 10.1152/ajpheart.00872.2009. 20852047 PMC3006283

[46] Dajnowiec D, Sabatini PJB, Van Rossum TC, Lam JTK, Zhang M, Kapus A, Langille BL. Force-Induced Polarized Mitosis of Endothelial and Smooth Muscle Cells in Arterial Remodeling. Hypertension 50: 255–260, 2007. doi: 10.1161/HYPERTENSIONAHA.107.089730. 17485596

[47] Eberth JF, Gresham VC, Reddy AK, Popovic N, Wilson E, Humphrey JD. Importance of pulsatility in hypertensive carotid artery growth and remodeling [Online]. Journal of Hypertension 27, 2009. https:/journals.lww.com/jhypertension/Fulltext/2009/10000/Importance_of_pulsatility_in_hypertensive_carotid.14.aspx 10.1097/HJH.0b013e32832e8dc8 PMC2783509 19584753

[48] Humphrey J, Eberth J, Dye W, Gleason R. Fundamental role of axial stress in compensatory adaptations by arteries. Journal of biomechanics 42: 1–8, 2009. 19070860 10.1016/j.jbiomech.2008.11.011 PMC2742206

[49] Humphrey JD, Schwartz MA. Vascular Mechanobiology: Homeostasis, Adaptation, and Disease. Annu Rev Biomed Eng 23: 1–27, 2021. doi: 10.1146/annurev-bioeng-092419-060810. 34255994 PMC8719655

[50] Matsumoto T, Hayashi K. Mechanical and Dimensional Adaptation of Rat Aorta to Hypertension. Journal of Biomechanical Engineering 116: 278–283, 1994. doi: 10.1115/1.2895731. 7799628

[51] Hu J-J, Ambrus A, Fossum TW, Miller MW, Humphrey JD, Wilson E. Time Courses of Growth and Remodeling of Porcine Aortic Media During Hypertension: A Quantitative Immunohistochemical Examination. J Histochem Cytochem 56: 359–370, 2008. doi: 10.1369/jhc.7A7324.2007. 18071063 PMC2326104

[52] Zhang Y. Structural and Mechanical Inhomogeneity in Arterial ECM: Implications for Physiology and Disease. In: Solid (Bio)mechanics: Challenges of the Next Decade: A Book Dedicated to Professor Gerhard A. Holzapfel, edited by Sommer G, Li K, Haspinger DCh, Ogden RW. Springer International Publishing, p. 73–94.

[53] Zou Y, Zhang Y. The orthotropic viscoelastic behavior of aortic elastin. Biomechanics and Modeling in Mechanobiology 10: 613–625, 2011. doi: 10.1007/s10237-010-0260-4. 20963623

[54] Yu X, Wang Y, Zhang Y. Transmural variation in elastin fiber orientation distribution in the arterial wall. Journal of the Mechanical Behavior of Biomedical Materials 77: 745–753, 2018. doi: 10.1016/j.jmbbm.2017.08.002. 28838859 PMC5696052

[55] Timmins LH, Wu Q, Yeh AT, Moore JE, Greenwald SE. Structural inhomogeneity and fiber orientation in the inner arterial media. American Journal of Physiology-Heart and Circulatory Physiology 298: H1537–H1545, 2010. doi: 10.1152/ajpheart.00891.2009. 20173046

[56] Pukaluk A, Wolinski H, Viertler C, Regitnig P, Holzapfel GA, Sommer G. Changes in the microstructure of the human aortic medial layer under biaxial loading investigated by multi-photon microscopy. Acta Biomaterialia 151: 396–413, 2022. doi: 10.1016/j.actbio.2022.08.017. 35970481

[57] Pukaluk A, Wolinski H, Viertler C, Regitnig P, Holzapfel GA, Sommer G. Changes in the microstructure of the human aortic adventitia under biaxial loading investigated by multi-photon microscopy. Acta Biomaterialia 161: 154–169, 2023. doi: 10.1016/j.actbio.2023.02.027. 36812954

[58] Murtada S-I, Kawamura Y, Weiss D, Humphrey JD. Differential biomechanical responses of elastic and muscular arteries to angiotensin II-induced hypertension. Journal of Biomechanics 119: 110297, 2021. doi: 10.1016/j.jbiomech.2021.110297. 33647550 PMC8044024

[59] McNulty M, Mahmud A, Spiers P, Feely J. Collagen type-I degradation is related to arterial stiffness in hypertensive and normotensive subjects. Journal of Human Hypertension 20: 867–873, 2006. doi: 10.1038/sj.jhh.1002015. 16598292

[60] Bashey RI, Cox R, McCann J, Jimenez SA. Changes in collagen biosynthesis, types, and mechanics of aorta in hypertensive rats. The Journal of laboratory and clinical medicine 113: 604–611, 1989. 2715682

[61] Qingyan Z, Xuejun J, Yanhong T, Zixuan D, Xiaozhan W, Xule W, Zongwen G, Wei H, Shengbo Y, Congxin H. Beneficial Effects of Renal Denervation on Pulmonary Vascular Remodeling in Experimental Pulmonary Artery Hypertension. Revista Española de Cardiología (English Edition) 68: 562–570, 2015. doi: 10.1016/j.rec.2014.11.022. 25804321

[62] Li P, Huang P-P, Yang Y, Liu C, Lu Y, Wang F, Sun W, Kong X-Q. Renal sympathetic denervation attenuates hypertension and vascular remodeling in renovascular hypertensive rats. Journal of Applied Physiology 122: 121–129, 2017. 27742806 10.1152/japplphysiol.01019.2015

[63] Wu L-L, Zhang Y, Li X-Z, Du X-L, Gao Y, Wang J-X, Wang X-L, Chen Q, Li Y-H, Zhu G-Q. Impact of Selective Renal Afferent Denervation on Oxidative Stress and Vascular Remodeling in Spontaneously Hypertensive Rats. Antioxidants 11: 1003, 2022. 35624870 10.3390/antiox11051003 PMC9137540

[64] Mahmud A, Feely J. Reduction in arterial stiffness with angiotensin II antagonist is comparable with and additive to ACE inhibition. American Journal of Hypertension 15: 321–325, 2002. doi: 10.1016/S0895-7061(01)02313-5. 11991217

[65] Harvey A, Montezano AC, Lopes RA, Rios F, Touyz RM. Vascular Fibrosis in Aging and Hypertension: Molecular Mechanisms and Clinical Implications. Canadian Journal of Cardiology 32: 659–668, 2016. doi: 10.1016/j.cjca.2016.02.070. 27118293 PMC4906153

[66] Patel RS, Al Mheid I, Morris AA, Ahmed Y, Kavtaradze N, Ali S, Dabhadkar K, Brigham K, Hooper WC, Alexander RW, Jones DP, Quyyumi AA. Oxidative stress is associated with impaired arterial elasticity. Atherosclerosis 218: 90–95, 2011. doi: 10.1016/j.atherosclerosis.2011.04.033. 21605864 PMC4059070

[67] Lefort C. A review of biomedical multiphoton microscopy and its laser sources*. Journal of Physics D: Applied Physics 50: 423001, 2017. doi: 10.1088/1361-6463/aa8050.

[68] Concannon J, Dockery P, Black A, Sultan S, Hynes N, McHugh PE, Moerman KM, McGarry JP. Quantification of the regional bioarchitecture in the human aorta. Journal of Anatomy 236: 142–155, 2020. doi: 10.1111/joa.13076. 31512228 PMC6904601

[69] Giudici A, Spronck B. The Role of Layer-Specific Residual Stresses in Arterial Mechanics: Analysis via a Novel Modelling Framework. Artery Research 28: 41–54, 2022. doi: 10.1007/s44200-022-00013-1.

[70] Rezakhaniha R, Agianniotis A, Schrauwen JTC, Griffa A, Sage D, Bouten CVC, van de Vosse FN, Unser M, Stergiopulos N. Experimental investigation of collagen waviness and orientation in the arterial adventitia using confocal laser scanning microscopy. Biomechanics and Modeling in Mechanobiology 11: 461–473, 2012. doi: 10.1007/s10237-011-0325-z. 21744269

[71] Touyz RM, Alves-Lopes R, Rios FJ, Camargo LL, Anagnostopoulou A, Arner A, Montezano AC. Vascular smooth muscle contraction in hypertension. Cardiovascular Research 114: 529–539, 2018. doi: 10.1093/cvr/cvy023. 29394331 PMC5852517

[72] Mulder J, Hökfelt T, Knuepfer MM, Kopp UC. Renal sensory and sympathetic nerves reinnervate the kidney in a similar time-dependent fashion after renal denervation in rats. American Journal of Physiology-Regulatory, Integrative and Comparative Physiology 304: R675–R682, 2013. doi: 10.1152/ajpregu.00599.2012. 23408032 PMC3627950

