# Supplementary material for: Renal denervation restores biomechanics of carotid arteries in a rat model of hypertension
Source: Res Sq. 2023 Sep 5:rs.3.rs-3273236. Preprint. [Version 1] doi: 10.21203/rs.3.rs-3273236/v1 (PMC10503847; doi:10.21203/rs.3.rs-3273236/v1)
Supplement: Supplement 1 [file NIHPPrs3273236v1-supplement-1.pdf]

Supplementary Files

This is a list of supplementary files associated with this preprint. Click to download.

- ratrenaldenervationsuppl.docx
